# Reduction of Fatigue and Musculoskeletal Complaints in Garment Sewing Operator through a Combination of Stretching Brain Gym^®^ and Touch for Health

**DOI:** 10.3390/ijerph18178931

**Published:** 2021-08-25

**Authors:** Lusi Ismayenti, Agus Suwandono, Hanifa Maher Denny, Bagoes Widjanarko

**Affiliations:** 1 Doctoral Program, Faculty of Public Health, Diponegoro University, Semarang 50275, Indonesia; suwandono49@gmail.com (A.S.); hanifadenny@live.undip.ac.id (H.M.D.); bagoes69@gmail.com (B.W.); 2 Departement of Epidemiology, Faculty of Public Health, Diponegoro University, Semarang 50275, Indonesia; 3 Departement of Occupational Safety and Health, Faculty of Public Health, Diponegoro University, Semarang 50275, Indonesia; 4 Departement of Health Promotion, Faculty of Public Health, Diponegoro University, Semarang 50275, Indonesia

**Keywords:** fatigue, musculoskeletal complaints, stretching, Brain Gym^®^, Touch for Health, garment-sewing operator

## Abstract

The purpose of this study was to assess the effect of using a combination of stretching and Brain Gym^®^(BG) + Touch for Health (TfH) movements to reduce fatigue and musculoskeletal complaints (MSCs) in garment-sewing operators. A quasi-experimental study was performed on 53 respondents with two sessions of stretching movements and BG + TfH movements of 5 min duration, three times a week for four weeks. Fatigue was measured using a reaction timer and MSCs were measured using a Nordic Body Map questionnaire. Wilcoxon and Mann–Whitney U tests were performed to examine the differences of pre/post and between the intervention group (IG) and control group (CG). A significant difference was found in IG for pre- and post-fatigue (*p* < 0.001) and MSCs (*p* < 0.001), while in CG there was no difference in fatigue (*p* = 0.200) and MSCs (*p* = 0.086). Significant differences were found between the IG and CG groups in terms of fatigue (*p* = 0.046), as well as in MSCs (*p* < 0.001). A significant decrease in MSCs per part body in IG was found on the left wrist, left hand, and left knee. The percentage of MSC severity decreased in all parts of the body, except the right shoulder, left elbow, and right thigh.

## 1. Introduction

The garment industry is a labor-intensive industry [1,2], forming 8.3% of the total trade in industrial materials in the world [3]. The production process is manual work (e.g., design development, assembly, sewing, and finishing). The garment-sewing operators’ work is in delimited rooms and their posture is restrained by both the visual and manual aspects of the task [4]. In addition, the garment-sewing operators’ work involves prolonged sitting positions and whole-day repetitive movement, and the rapid pace of the work leads to risk of fatigue and musculoskeletal disorders (MSDs) [5,6,7,8,9]. Fatigue has contributed to the occurrence of MSDs and is still a problem for ergonomics researchers around the world [10], affecting mental and physical health [11,12], increasing the frequency of workplace accidents, and decreasing productivity [13]. Characteristic individual, psychosocial, and environmental factors also affect MSDs [14]. Prolonged fatigue without adequate rest can increase the severity of MSDs [15].

The most prevalent garment-sewing operator musculoskeletal complaints involve muscles of the neck, shoulders, arms, elbows, wrists [16], hands, fingers, back, waist, and lower extremities such as the knee [17] and the right leg [18,19,20]. Thus, it is important to create a preventive program for garment-sewing operators. Several preventative MSD programs have been provided, such as modified work stations [10,16], modified production processes [21], ergonomics training for workers [22], policies to reduce exposure to risk factors, changes in working methods and procedures such as providing time off for resting [23], work rotation and training [16], and the provision of personal protective equipment (PPE) such as anti-vibration gloves and vibration-absorbing soles on the feet [24]. Participatory ergonomics programs have been implemented, but have not fully reduced fatigue and MSDs [25,26,27].

In a previous study, it was reported that physical exercise can control the risk factors of fatigue [28,29] and MSDs [30,31,32,33,34,35,36,37,38,39,40]. The types of physical exercise used were physical exercise [30], stretching [31,32], resistance training [28,33], strength training [34], and a combination of stretching and games [30]. In addition, a combination of stretching, resistance, and strength training provides the best results [33,41]. Physical exercise has various durations and the frequency of intervention times varies: resistance exercises once a day for 20 min, three times a week for 4 months [28]; 1-h low-intensity running sessions three times a week for a period of six consecutive weeks [29]; 36 different stretches once every 6 min, with stretches lasting 10 to 15 s for 15–17 workdays [31]; a worksite-based physical activities program once a day, lasting 10 min, for 12 months [35]; 10 min daily exercise session for 2 months [36]; physical exercise for 20 min, three times a week for 12 months [37]; resistance training lasting 2 and 12 min, five times a week for 10 weeks [33]; three strength training interventions with different frequencies and durations (1 h once a week, 20 min three times a week, and 7 min nine times a week) for 2 weeks [34]; stretching two times/week for 5–15 min, lasting 6 weeks [38]; exercise training for 10–15 min, three times a week for 11 weeks [39]; exercise two times a week for 3 months [30]; and stretching exercises lasting 10–15 min, two times a day for 4 weeks [32].

Brain Gym (BG) is a simple stretching motion to lengthen muscles in order to maintain balanced mind–body coordination [42]. It is also used to reduce the mental fatigue of workers due to the high level of accuracy required in their work, and to relax muscle and tendon tension due to brainstem reflexes. This results in feelings of relaxation through a rearrangement of proprioceptors [43,44], and increases alertness by increasing the reaction times [45]. This intervention has also been proven to accelerate the blood circulation in the body, especially the supply of oxygen to brain cells [46]. This method can be applied to garment-sewing operators whose work requires a high accuracy. Thus, it will create energy flow and provide balance to the muscle, or ensure healthy muscle [47]. The four BG movements selected as having benefits for garment-sewing operators are as follows: increasing the strength and balance of the neck and shoulder muscles to reduce the forward-leaning posture (the owl movement), lengthening the upper chest and shoulder muscles, helping to balance the chest and back muscles, and relaxing the fingers. This increases focus, making it easier to coordinate the hands, eyes, and skills using equipment (arm activation movement); restores the ability to realize the position of the muscles (increased proprioception); and stabilizes and coordinates the lower back, hips, and legs, increasing the concentration and attention required for conscientious work and leading to full-body relaxation and ease in prolonged sitting (the grounder movement). It also synchronizes the hips, hamstrings, and lower back; relaxes the neck; improves focus; and leads to a more active posture and increased body comfort during prolonged sitting periods (the gravity glider movement) [43].

Touch for Health (TfH) is generally used as a therapy, but also can be performed in stretching movements. Brain Gym and TfH are new methods used in physical stretching to lengthen and strengthen muscles. The selection of BG and TfH is based on their simplicity and ease of movement, as well as the lack of requirements for extra time and energy. These movements were chosen to minimize the risk factors for pain in the garment-sewing operator, from the neck muscles to the feet. The eight TfH movements shown to garment-sewing operators can help overcome problems around the shoulder muscles and strengthen the arm and brain muscles (supraspinatus muscle); flex the spine from the thoracic vertebrae to the lumbar (teres major muscle); support respiratory and lung function; strengthen the scapula (serratus anterior muscle); strengthen the heart and arms (subscapular muscle); optimize kidney function and maintain the lumbar curvature of the spine (psoas muscle); make it easier to move the arm inward, rotate the arm, and move it forward (pectoralis major sternalis muscle); optimize pancreatic spleen function; maintain an erect back (latissimus dorsi) and bladder; and stretch the ankle muscles (peroneus muscle) [48].

Garment-sewing operators work 8 h per day with a break time of 1 h, after sewing continuously for 4 h, which causes muscle tension. Activities that require long periods of physical work require rest to recover energy. Pulat [49] explained that giving an operator a break time of 10 min per hour can help them recover from fatigue. Meanwhile, sewing jobs in the garment industry are based on daily production targets, so garment-sewing operators find it difficult to take break time. Lacaze at al. reported [36] that giving a rest break of 10 min is no better than stretching for the same time to reduce fatigue and musculoskeletal discomfort. 

Several studies on stretching to reduce fatigue and MSDs have been carried out on workers in sitting positions, such as workers in call centers [36], office workers [30,32,33,34,37,38,39,41], and bus drivers [50]. However, there have been no studies yet on stretching focusing on garment-sewing operators. This study provides a combination of stretching movements and BG + TfH movements. Therefore, this study aims to assess the effect of 5-min sessions (two-session) of an intervention technique to reduce fatigue and MSCs in garment-sewing operators by implementing a combination of stretching and BG + TfH movements.

## 2. Materials and Methods

This study used a quasi-experimental design with the intervention group (IG) and the control group (CG). The garment industry was used in this study: the garment industry was willing to permit visits and accept direct interaction with respondents during the COVID-19 pandemic. A total of 53 garment-sewing operators were studied, from the garment industry in Karanganyar Regency, Central Java Province, Indonesia.

### 2.1. Subject

The garment-sewing operators who participated in this study were female, not pregnant, did not smoke, had not experienced trauma to the bones and muscles, did not take anti-depressant drugs, and used the same machine and sewing materials. Fifty-three garment sewing operators were selected and given an informed consent sheet. Their willingness to be a respondent was assessed, and their personal data were provided as follows: age, years of service, body mass index, blood pressure, and pulse. The operators participated voluntarily. The division of the garment-sewing operator group was carried out by distributing lottery papers assigning respondents to the IG and CG. This resulted in the following group sizes: IG = 27 and CG = 26. The researchers conducting the intervention did not know who was included in the IG or CG.

### 2.2. Intervention Program

**Figure 1:**
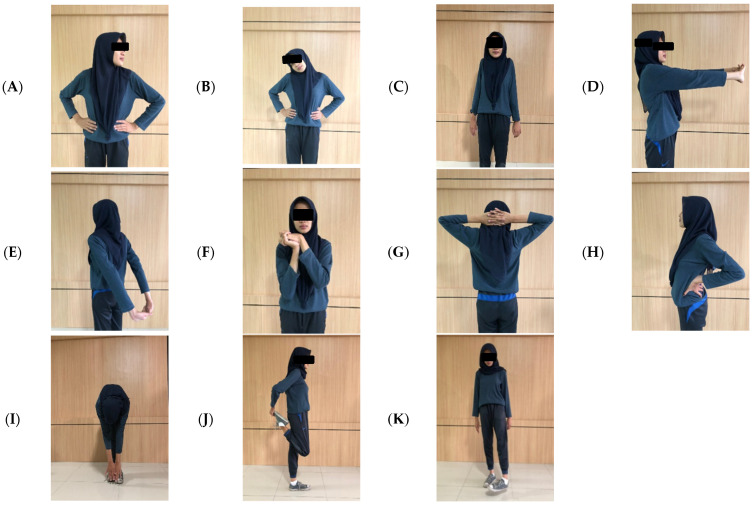
(**A**) Neck and shoulder stretches; (**B**) neck, shoulders, and arm stretches; (**C**) shoulder stretch; (**D**) arm and upper back stretches; (**E**) shoulders, arm, and chest stretches; (**F**) hands, wrists, finger, and forearm stretches; (**G**) upper back stretch; (**H**) back, shoulder, and arm stretches; (**I**) low back, hips, groin, and hamstring stretches; (**J**) lower leg and leg joint stretches; (**K**) lower limb and ankle stretches.

**Figure 2:**
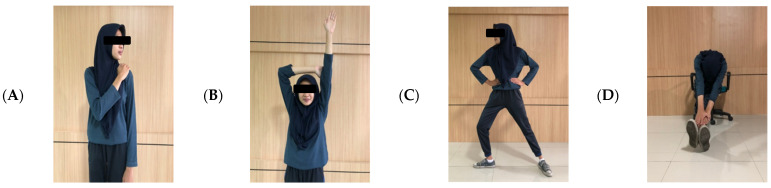
(**A**) The owl movement; (**B**) arm activation movement; (**C**) grounder movement; (**D**) gravity glider movement.

**Figure 3:**
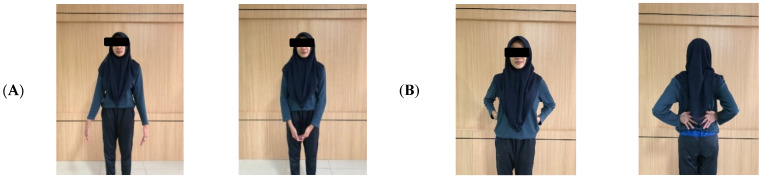

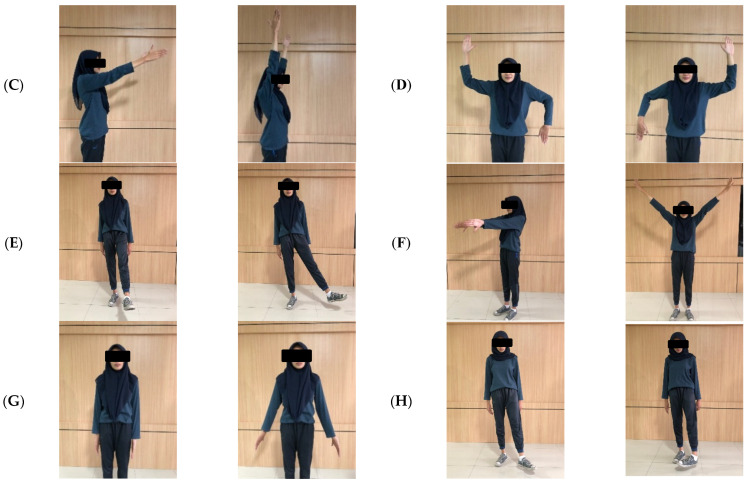
(**A**) Supraspinatus muscle; (**B**) teres major muscle; (**C**) serratus anterior muscle; (**D**) subscapular muscle; (**E**) psoas muscle; (**F**) pectoralis major sternalis muscle; (**G**) latissimus dorsi; (**H**) peroneus muscle.

The interventions lasted 5 min for each session (two-session), and took place three times a week for 4 weeks. In the first session, a stretching movement was carried out 2 h after working, before the break time. This consisted of 11 light stretches used to stretch stiff muscles with a count of 2 × 8 (Figure 1). The second session consisted of four BG (Figure 2) + 8 TfH (Figure 3) movements for 5 min, which were specially selected according to the garment-sewing operator’s work. Brain gym and TfH were performed 2 h after breaktime, with a slow count of 1 × 8.

The intervention was performed three times a week for four weeks. The intervention group was guided by a video and was directly monitored by the researcher to maintain the accuracy of the movement. During the intervention period, the IG conducted an intervention program, while the CG continued to work on sewing. The intervention program was divided into stretching and BG + TfH movements, designed and combined by the researcher. The movements were structured and adapted according the garment-sewing operators’ work, to reduce muscle tension from the neck muscles to the feet.

#### 2.2.1. Stretching Movement

The stretching movements focused on stretching the neck, shoulders, upper back, chest, hips, arms, forearm, hands, wrists, fingers, groin, hamstring, lower leg, leg joint, lower limb, and ankle [51]. Every movement was applied in 2 × 8 counts.

#### 2.2.2. Combination BG + TfH Movements

The four BG movements were owl movement, horse tide movement, arm activation movement, and gravity glider movement [43]. The eight TfH movements were chosen to move the supraspinatus muscle, teres major, serratus anterior, subscapularis, psoas, pectoralis major sternalis, latissimus dorsi, and peroneus [52].

The Brain Gym was designed with four movements, consisting of owl movement, arm activation movement, grounder movement, and gravity glider movement. The owl movement consists of grasping the top of one shoulder with the opposite hand and squeezing the muscles firmly. Slowly, the head is turned to look back over the shoulder. Draw the shoulders back, open the chest, and exhale for a count of eight. Continue to squeeze the shoulder as the head is turned to look over the other shoulder, “hooting” as the chest is opened again. “Hoot” again and drop the head toward the chest, keeping the chin tucked in and relaxing the shoulder down and back. The arm activation movement consists of standing with feet parallel and shoulder-width apart. Raise one arm above the head, using the opposite hand to hold it next to the ear. Exhale gently through pursed lips, while activating the muscles isometrically by pushing the raised arm against the other hand. The grounder movement consists of standing with the feet a leg length apart, and hands on the hips. Then, turn the head and right foot to the right, keeping the left foot pointed straight ahead. The torso and pelvis face squarely forward; the head, the bending knee, and the foot of the bent leg face to the side. Bend the right knee and slowly exhale, allowing the knee to extend to the middle of the right foot. Inhale and straighten that leg, then return to an upright position. The gravity glider movement consists of sitting up comfortably on the edge of a sturdy chair and extending the feet, resting on the floor. Keeping the knees relaxed, bend forward and reach out to the front, letting the head move freely. Then, let gravity take over and the arms glide down, and exhale. Inhale upon moving back up.

Touch for Health consists of the following: Supraspinatus muscle movements, where both arms move towards the center in a V shape. Teres major muscles movements, with hands on hips and elbows slowly flapping like a chicken, while the shoulders remain steady. Serratus anterior muscle movements consist of the hand positioned straight upward while the thumb moves over the shoulder. Move slowly while taking a deep breath, and then lower the arm while exhaling. Subscapular muscle movements begin with the right and left upper arms in line with the shoulders, with the right forearm above and the left forearm below. Then, slowly swing the arms up and down. In the psoas muscle movements, the hips are steady and the soles of the feet are tilted outward. Legs swing 45° forward, sideways, and then closed. Repeat in a waltz, (count 1-2-3) forward, sideways, close, and forward again. Perform this twice, while alternating the left and right leg. The pectoralis major sternalis muscle movements are performed slowly, like swimming, but the hands are lifted up and out, while saying, “Hore”. The latissimus dorsi movement begins with palms facing outward. Lift the hand outward slowly, like a penguin’s wing. The peroneus muscle movement is similar to kicking a ball, with the sole of the foot moving slowly inward.

### 2.3. Instrument and Measurement Protocol

Fatigue was measured using a reaction timer machine (Lakassidaya L-77/EP-354-L77-103, PT. Sigma Global Med, Indonesia, 1 s/0.1 millisecond) with an accuracy of ±0.0014 s (k = 2). The garment-sewing operators pressed the mouse button while watching the signal light flash as quickly as possible, using the dominant index finger to avoid arm and elbow motion changes. The signal light was switched on with a varied tempo given by the enumerator. The signal light was shown 20 times to the garment sewing operator, and the results were recorded directly by the enumerator. The examination results in numbers 1–5 and 16–20 were omitted, because they were considered to be within the emerging adjustment and saturation levels. Reaction timer evaluation was used to determine changes in fatigue in garment-sewing operators by comparing the measurement results before and after the intervention.

The level of MSCs was assessed by the Nordic Body Map (NBM) questionnaire [53]. The Nordic Body Map questionnaire consists of statements regarding 28 parts of the body that experience pain, with a severity of 0 indicating there is no pain at all; 1 indicating there is pain in a certain muscle area, but it does not interfere with work; 2 indicating muscle pain that has disturbed workers and forces a break in work to reduce pain; and 3 indicating very annoying pain that does not go away even after resting [54]. Garment-sewing operators completed the NBM questionnaire individually, but under the supervision of the enumerators, so that if they found difficulties in answering, they could ask directly. Fatigue and MSCs measurements were carried out before and after the intervention on the IG and CG.

### 2.4. Statistical Analysis

The results of the fatigue evaluation and MSCs were compiled into ratio data. The IBM SPSS Statistics 22 (IBM, Armonk, NY, USA) program was used for the statistical evaluation and the measured values of all variables were calculated as the mean, median, min, max, interquartile range (IR), and standard deviation (SD). The variables showed there was not a normal distribution based on the Shapiro-Wilk tests, and the Levene test was applied for homogeneity variances. The differences in the results of pre- and post-fatigue and MSDs were tested using the Wilcoxon test. The *p*-value was *p* < 0.05, and a significant difference was seen between the pre- and post-results in each group. The Mann–Whitney U test determined the difference in fatigue results and MSDs between the intervention and control groups. A *p*-value of <0.05 indicates a difference between the IG and CG.

### 2.5. Ethics Approval 

Garment-sewing operators were given an approval sheet to ascertain whether they were willing to be a respondent or not, because this research was voluntary. This research was approved by the Health Research Ethics Committee of Sebelas Maret University with the identity number 177/UN27.06.6.1/KEPK/EC/2020.

## 3. Results

The 53 garment-sewing operators recruited in this study met the established criteria. Random sampling was used to divide the intervention (IG: 27) and control (CG: 26) groups. The characteristics of the respondents are presented in Table 1. No significant difference was seen in the homogeneity test between respondents’ characteristics in the intervention group and the control group (*p* > 0.05).

### 3.1. Variation in Fatigue

The results obtained after the evaluation of the fatigue pre- and post-measurements are shown in Table 2. After the intervention, an evaluation of the results showed a significant difference in the IG (*p* < 0.001), and there was a decrease in the mean between pre- and post-values. In contrast, there was no difference in the CG (*p* = 0.200); an increase was seen between the pre- and post-scores. The intervention and control groups showed significant differences (*p* = 0.046).

### 3.2. Variation in Musculoskeletal Complaints

Musculoskeletal complaints regarding IG decreased, and there was a difference between the pre–post values (*p* < 0.001), whereas CG without intervention showed an increase, and there was no difference (*p* = 0.106). The evaluation results of the comparison between IG and CG showed a significant difference (*p* < 0.001) (Table 3).

Musculoskeletal complaints per body part (pre-post) are shown in Table 4. After the intervention program, a significant decrease in the IG was seen for the left wrist (*p* = 0.008), left hand (*p* = 0.046), and left knee (*p* = 0.035), and was constant on the right shoulder (*p* = 1.000), right lower arm (*p* = 1.000), and left elbow (*p* = 1.000). A significant increase in the CG was seen on the bottom (*p* = 0.032), left elbow (*p* = 0.011), right elbow (*p* = 0.021), left upper arm (*p* = 0.032), right upper arm (*p* = 0.005), and left ankle (*p* = 0.013), and was constant on the left shoulder (*p* = 0.971), right shoulder (*p* = 1.000), waist (*p* = 0.976), and left wrist (*p* = 0.184).

**Figure 4:**
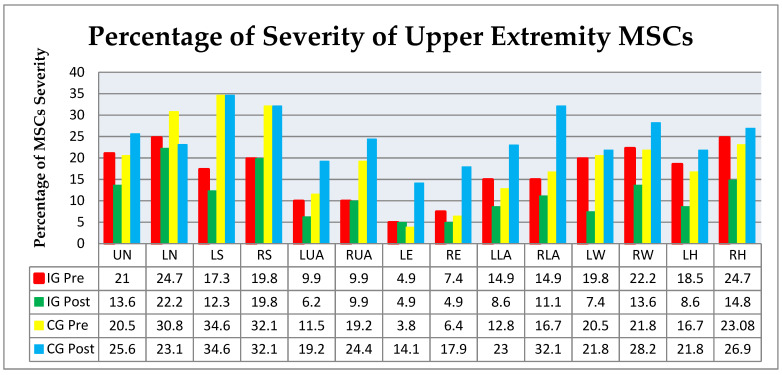
Percentage of upper extremity MSC severity. IG Pre—intervention group before the intervention; IG Post—intervention group after the intervention; CG Pre—control group before the intervention; CG Post—control group after the intervention. Musculoskeletal system: UN (upper neck), LN (lower neck), RS (right shoulder), LS (left shoulder), LUA (left upper arm), RUA (right upper arm), LE (left elbow), RE (right elbow), LLA (left lower arm), RLA (right lower arm), LW (lower wrist), RW (right wrist), LH (left hand), and RH (right hand).

There was severity in the upper extremity between the IG and CG (pre-post) are shown in Figure 4; the largest decrease in severity of MSCs with the IG was seen in the lower wrist (−12.4%), right and left hand (−9.9%), right wrist (−8.6%), upper neck (−7.4%), and left lower arm (−6.5%). The largest increase in the CG was seen in the right elbow (+11.5%), left elbow (+10.3%), left lower arm (+10.2%), and left upper arm (+7.7%), while a decrease also accrued in the lower neck (−7.7%).

**Figure 5:**
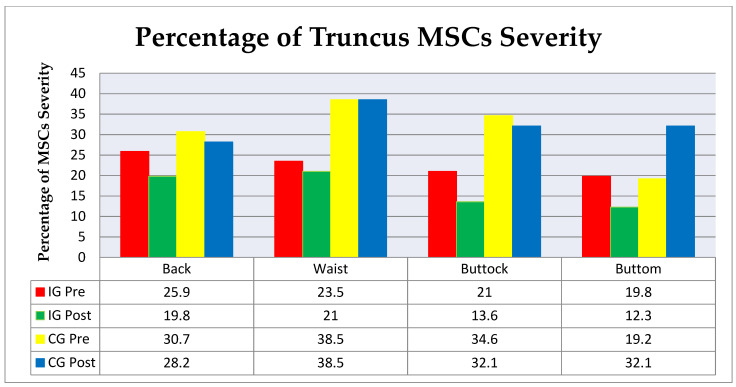
Percentage of truncus MSC severity. IG Pre—intervention group before the intervention; IG Post—intervention group after the intervention; CG Pre—control group before the intervention; CG Post—control group after the intervention.

Similar to the upper extremity, a decreased percentage of truncus MSCs severity in the IG extremes was seen in the bottom (−7.5%), buttock (−7.4%), and back (−6.1%). The control group had the highest increase (+12.9%), a decrease in the back and buttock (−2.5%), and constant values in the waist (Figure 5).

**Figure 6:**
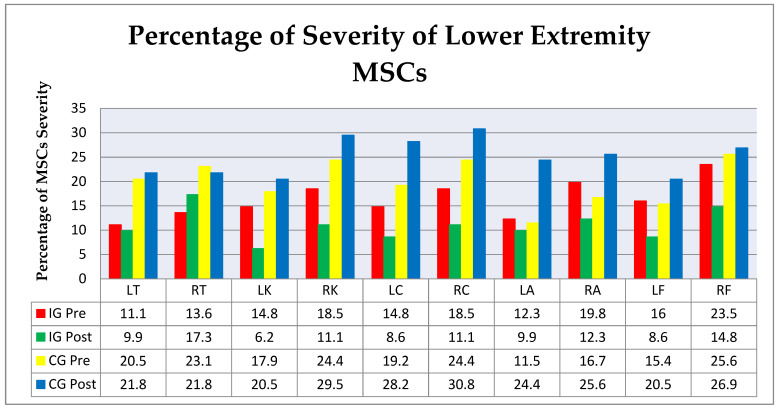
Percentage of lower extremity MSC severity. IG Pre—intervention group before the intervention; IG Post—intervention group after the intervention; CG Pre—control group before the intervention; CG Post—control group after the intervention. Musculoskeletal system: LT (left thigh), RT (right thigh), LK (left knee), RK (right knee), LC (left calf), RC (right calf), LA (left ankle), RA (right ankle), LF (left foot), and RF (right foot).

The comparison of severity in the lower extremity between the IG and CG (pre–post) in Figure 6, showed that the largest decrease in the severity of MSCs with lower IG extremities occurred in the right foot (−8.7%), left knee (−8.6%), right ankle (−7.5%), right knee, right calf, and right foot (−7.4%). The largest increase in CG was seen in the left ankle (+12.9%), left calf (+9%), right ankle (+8.9%), and right calf (+6.4%), while a decrease also occurred in the right thigh (−1.3%).

## 4. Discussion

The garment-sewing operator’s work week covers 45 h (8 h a day on weekdays and 5 h on Saturday), with a break time of an hour. They work constantly for hours without a coffee break due to the daily production target, which requires that they remain focused. It is difficult for them to take additional break times during working hours.

This study assessed the effect of applying a combination of stretching and BG + TfH movements for 5-min sessions for 4 weeks, and found that the results significantly reduced fatigue. A previous study also demonstrated that appropriately designed and supervised 10 min daily stretching programs for 2 months during the work shift are more effective than rest breaks at reducing both musculoskeletal discomfort and physical and mental fatigue in call-center workers [36]. This study separated the intervention program into two sessions, to allow workers to refresh after working constantly without a coffee break.

A significant reduction in MSCs was seen in the group who received the intervention program. The body parts that experienced a significant decrease were the left wrist, left hand, and left knee. This study shows that sewing work uses dynamic movements that are more dominant in the right muscles from the upper extremities [16] to the lower extremities [17], thus providing a balanced stretch and strengthening the left and right muscles [43,48,51]. These results were in line with previous studies showing the benefit of stretching for this condition; for example, a prior study also provided worksite physical activities programs to female workers, focusing on spine, upper limbs, and lower limbs [35].

The percentage reductions in MSC severity occurred in the left wrist, right and left hand, right wrist, right ankle, right knee, right calf, and right foot. The increasing and constant results in certain muscles are due to their being dominant muscles used in working, such as the left elbow holding the fabric position, the right thigh keeping and adjusting the pedal speed to synchronize it with the hand positioning the fabric, and the right upper arm, which works more dominantly [16,19,20]. The position of the garment sewing operators, with their head bowed while sewing, results in neck and lower neck pain. The positioning of the head, which is not in the straight line of the spine, prompts neck pain [8], and increasing the endurance of the neck and shoulder muscles can prevent neck pain [40]. In addition, the prolonged sitting position of a garment-sewing operator promotes complaints of pain in the neck, lower back, and knee [9].

The duration and frequency of program interventions from the study of Marangoni et al. [31], with stretching once every six minutes, lasting 10 to 15 s, every day for 15–17 days, can reduce MSDs pain in office workers without changing the work layout, which is considered to disturb the concentration of workers due to the interrupted work time. In this study, the combination of stretching and BG + TfH movements for 5 min in two sessions three times a week, carried out at the side of the desk, did not interfere with work, refreshed workers from monotonous work [6,7,8], and reduced musculoskeletal complaints.

Most other studies were carried out over a longer duration [28,29,30,39]; this study showed a decrease in complaints of general musculoskeletal fatigue over the four-week intervention. Previous research, conducted by Lee et al. [50], showed a significant decrease in musculoskeletal symptoms in the neck and shoulders after self-stretching exercise for 4 weeks.

Most studies were interested in strengthening or resistance exercises, including the study of Santos et al. [28] and Andersen et al. [33,34,37]; they emphasized that strengthening exercises and specific resistance training of the upper extremity can relieve musculoskeletal pain symptoms in office workers. However, this study looked at the effect of combination stretching and BG + TfH movements that can reduce musculoskeletal complaints without using assistive devices, the movements are not a high risk if you experience the wrong movements, the movements are easy and light.

This study has several limitations. First, the small sample size of 53 subjects was the result of difficulties in finding respondents during the COVID-19 pandemic period. Therefore, the results are difficult to generalize and interpret. Second, the length of the intervention period was only 4 weeks; a longer intervention study is needed to confirm the findings and long-term effects. Third, the difficulty of finding time for the intervention and data collection due to the high production targets must be resolved.

## 5. Conclusions

In conclusion, by comparing the two groups, it was found that the combination of stretching and BG + TfH movements can reduce fatigue and musculoskeletal complaints. Decreased MSCs per body part were found on the left wrist, left hand, and left knee. The percentage of MSCs’ severity decreased in the upper extremities (except for right shoulder and left elbow which were constant), truncus, and lower extremities (except for the increased right thigh).

There have been no other studies to date on the provision of stretching and BG + TfH movement interventions for garment-sewing operators. Performing easy and light movements for 5 min twice a day can be beneficial for sewing operators, who work constantly for hours without a coffee break due to their daily production targets, which require them to remain focused. This study is considered a guide for future interventions with the same objective, with a larger sample and longer intervention time.

## Figures and Tables

**Table 1:** Distribution of garment-sewing operator characteristics (*n* = 53).

Variable		IG (*n* = *27*)	CG (*n* = *26*)	*P*
Age (year)		31.33 ± 10.52	32.12 ± 9.04	0.315
Working period (year)		7.37 ± 7.23	5.09 ± 6.06	0.270
Pulse (bpm)		97.37 ± 14.80	91.08 ± 13.71	0.706
Weight (kg)		55.78 ± 11.25	53.12 ± 12.80	0.259
Height (cm)		152.31 ± 4.62	152.42 ± 5.16	0.540
Body mass index (BMI) (kg/m^2^)		24.05 ± 4.94	22.91 ± 5.53	0.176
Variable		*n*	Frequency	Percentage (%)
Education	Elementary School	53	3	5.6
	Middle School		20	37.7
	High School		30	56.7
Smoke	Yes	53	0	0
	No		53	100

Data are reported as mean ± SD; IG—intervention group; CG—control group.

**Table 2:** Fatigue before and after the intervention. Data are reported as median ± interquartile range.

	Fatigue			
	Pre	Post	*p* ^0^	*p* ^1^
IG	319.16 ± 197	279.79 ± 155	<0.001	0.046
CG	257.7 ± 121	260.57 ± 89	0.200	

Note: Pre—data before intervention; Post—data after intervention; ^0^—Wilcoxon test; ^1^—Wilcoxon Mann−Whitney U test.

**Table 3:** MSCs before and after the intervention. Data are reported as median ± interquartile range.

	MSCs			
	Pre	Post	*p* ^0^	*p* ^1^
IG	15 ± 15	9 ± 12	<0.001	<0.001
CG	16.5 ± 11	20 ± 16	0.106	

Note: Pre—data before intervention; Post—data after intervention; ^0^—Wilcoxon test; ^1^—Wilcoxon Mann−Whitney U test.

**Table 4:** Musculoskeletal complaints per body part. Data are expressed mean ± SD.

	Intervention Group		*p*	Mean Diff	Control Group		*p*	Mean Diff
	Pre Mean ± SD (Min − Max)	Post Mean ± SD (Min − Max)			Pre Mean ± SD (Min − Max)	Post Mean ± SD (Min − Max)		
Upper Neck	0.63 ± 0.9 (0.0–4.0)	0.41 ± 0.6 (0.0–2.0)	0.317	−0.22	0.62 ± 0.6 (0.0–2.0)	0.77 ± 0.6 (0.0–2.0)	0.248	+0.15
Lower Neck	0.74 ± 0.8 (0.0–3.0)	0.67 ± 0.6 (0.0–2.0)	0.635	−0.07	0.92 ± 0.6 (0.0–2.0)	0.69 ± 0.5 (0.0–2.0)	0.153	−0.23
Left Shoulder	0.52 ± 0.7 (0.0–2.0)	0.37 ± 0.6 (0.0–2.0)	0.206	−0.15	1.04 ± 0.7 (0.0–2.0)	1.04 ± 0.8 (0.0–2.0)	0.971	0
Right Shoulder	0.59 ± 0.7 (0.0–2.0)	0.59 ± 0.6 (0.0–2.0)	1.000	0	0.96 ± 0.7 (0.0–2.0)	0.96 ± 0.7 (0.0–2.0)	1.000	0
Left Lower Arm	0.30 ± 0.7 (0.0–2.0)	0.19 ± 0.4 (0.0–1.0)	0.380	−0.11	0.35 ± 0.6 (0.0–2.0)	0.58 ± 0.6 (0.0–2.0)	0.109	+0.23
Back	0.78 ± 0.7 (0.0–2.0)	0.59 ± 0.6 (0.0–2.0)	0.215	−0.19	0.92 ± 0.8 (0.0–2.0)	0.85 ± 0.7 (0.0–2.0)	0.617	−0.07
Right Lower Arm	0.30 ± 0.6 (0.0–2.0)	0.30 ± 0.4 (0.0–1.0)	1.000	0	0.58 ± 0.6 (0.0–3.0)	0.73 ± 0.6 (0.0–2.0)	0.206	+0.15
Waist	0.70 ± 0.8 (0.0–2.0)	0.63 ± 0.6 (0.0–2.0)	0.635	−0.07	1.20 ± 0.7(0.0–3.0)	1.20 ± 0.7 (0.0–2.0)	0.976	0
Buttock	0.63 ± 0.7 (0.0–2.0)	0.41 ± 0.6 (0.0–2.0)	0.175	−0.22	1.00 ± 0.8 (0.0–2.0)	0.96 ± 0.7 (0.0–2.0)	0.771	−0.04
Bottom	0.59 ± 0.8 (0.0–3.0)	0.37 ± 0.6 (0.0–2.0)	0.109	−0.22	0.58 ± 0.7 (0.0–1.0)	0.96 ± 0.9 (0.0–2.0)	0.032 *	+0.38
Left Elbow	0.15 ± 0.6(0.0–3.0)	0.15 ± 0.5 (0.0–2.0)	1.000	0	0.12 ± 0.3 (0.0–2.0)	0.42 ± 0.6 (0.0–2.0)	0.011 *	+0.30
Right Elbow	0.22 ± 0.6(0.0–3.0)	0.15 ± 0.5 (0.0–2.0)	0.680	−0.07	0.19 ± 0.6 (0.0–2.0)	0.54 ± 0.7 (0.0–2.0)	0.021 *	+0.35
Left Upper Arm	0.44 ± 0.8 (0.0–3.0)	0.26 ± 0.6 (0.0–2.0)	0.059	−0.18	0.39 ± 0.6 (0.0–2.0)	0.77 ± 0.9 (0.0–3.0)	0.032 *	+0.38
Right Upper Arm	0.44 ± 0.7 (0.0–2.0)	0.33 ± 0.6 (0.0–2.0)	0.257	−0.11	0.50 ± 0.6 (0.0–2.0)	0.96 ± 0.8 (0.0–2.0)	0.005 *	+0.46
Left Wrist	0.59 ± 0.8 (0.0–3.0)	0.22 ± 0.5 (0.0–2.0)	0.008 *	−0.37	0.62 ± 0.8(0.0–2.0)	0.65 ± 0.7 (0.0–2.0)	0.184	0
Right Wrist	0.67 ± 0.8 (0.0–3.0)	0.41 ± 0.5 (0.0–1.0)	0.106	−0.16	0.65 ± 0.8 (0.0–2.0)	0.85 ± 0.9 (0.0–3.0)	0.190	+0.20
Left Hand	0.56 ± 0.8 (0.0–2.0)	0.26 ± 0.5 (0.0–2.0)	0.046 *	−0.30	0.50 ± 0.7 (0.0–2.0)	0.65 ± 0.7 (0.0–2.0)	0.331	+0.15
Right Hand	0.74 ± 0.8 (0.0–2.0)	0.44 ± 0.5 (0.0–1.0)	0.073	−0.30	0.69 ± 0.7 (0.0–2.0)	0.81 ± 0.7 (0.0–2.0)	0.366	+0.12
Left Thigh	0.33 ± 0.7 (0.0–2.0)	0.30 ± 0.5 (0.0–2.0)	0.705	−0.03	0.62 ± 0.6 (0.0–2.0)	0.58 ± 0.8 (0.0–3.0)	0.763	−0.04
Right Thigh	0.41 ± 0.6 (0.0–2.0)	0.52 ± 0.5 (0.0–2.0)	0.257	+0.11	0.69 ± 0.6 (0.0–2.0)	0.65 ± 0.7 (0.0–2.0)	0.763	−0.04
Left Knee	0.44 ± 0.8 (0.0–3.0)	0.19 ± 0.4 (0.0–1.0)	0.035 *	−0.25	0.54 ± 0.7 (0.0–2.0)	0.62 ± 0.7(0.0–2.0)	0.564	+0.08
Right Knee	0.56 ± 0.8 (0.0–3.0	0.33 ± 0.6 (0.0–2.0)	0.130	−0.23	0.73 ± 0.8 (0.0–1.0)	0.89 ± 0.9 (0.0–3.0)	0.372	+0.01
Left Calf	0.44 ± 0.7 (0.0–2.0)	0.26 ± 0.4 (0.0–1.0)	0.059	−0.18	0.58 ± 0.5 (0.0–2.0)	0.85 ± 0.8(0.0–2.0)	0.151	+0.27
Right Calf	0.56 ± 0.8 (0.0–3.0)	0.33 ± 0.5 (0.0–1.0)	0.109	−0.23	0.73 ± 0.5 (0.0–2.0)	0.92 ± 0.8 (0.0–2.0)	0.272	+0.19
Left Ankle	0.37 ± 0.7 (0.0–2.0)	0.30 ± 0.6 (0.0–2.0)	0.414	−0.07	0.35 ± 0.6 (0.0–2.0)	0.73 ± 0.7 (0.0–2.0)	0.013 *	+0.38
Right Ankle	0.59 ± 0.8 (0.0–3.0)	0.37 ± 0.6 (0.0–2.0)	0.058	−0.22	0.50 ± 0.6 (0.0–2.0)	0.77 ± 0.8 (0.0–3.0)	0.124	+0.22
Left Foot	0.48 ± 0.9 (0.0–4.0)	0.26 ± 0.5 (0.0–2.0)	0.132	−0.22	0.46 ± 0.7 (0.0–2.0)	0.62 ± 0.7 (0.0–2.0)	0.206	+0.16
Right Foot	0.70 ± 1.0 (0.0–4.0)	0.44 ± 0.6 (0.0–2.0)	0.083	−0.26	0.77 ± 0.7(0.0–2.0)	0.81 ± 0.7 (0.0–2.0)	0.803	+0.04

Note: Pre—data before intervention; Post—data after intervention; *p*—Wilcoxon test; * *p* < 0.05.

## Data Availability

All data generated during the study are available from: appropriate author upon reasonable request.
